# High Revision Rate After Transphyseal ACL Reconstruction in Skeletally Immature Patients

**DOI:** 10.3390/jpm14121129

**Published:** 2024-11-29

**Authors:** Benjamin Bartek, Tobias Jung, Theresa Lackner, Imke Schatka, Clemens Gwinner, Thula Walter-Rittel

**Affiliations:** 1 Center for Musculoskeletal Surgery, Charité-University Medicine Berlin, 10117 Berlin, Germany; 2 Clinic for Pediatric Orthopedics and Pediatric Traumatology, Klinikum Emil von Behring, 14165 Berlin, Germany; 3 Center for Diagnostic and Interventional Radiology and Nuclear Medicine, Charité-University Medicine Berlin, 10117 Berlin, Germany

**Keywords:** ACL reconstruction, transphyseal drilling, growth plates, physis, adolescent

## Abstract

**Objectives:** There remains considerable debate regarding the optimal management of anterior cruciate ligament (ACL) injuries in skeletally immature patients. This study aims to evaluate the clinical outcomes of transphyseal ACL reconstruction in patients with open growth plates. **Methods:** This retrospective study included skeletally immature patients with full-thickness ACL tears and confirmed open physis. ACL reconstructions were performed using a four-strand semitendinosus autograft, with an additional gracilis tendon graft if needed. The surgical technique emphasized tibial and femoral physeal-sparing tunnel placement to minimize disruption of the growth plates. Clinical assessment included measurements for limb length discrepancy, knee stability, and growth disturbances. Functional outcomes were evaluated using IKDC 2000, Lysholm, and KOOS scores, while ligament stability was assessed with KT-1000 arthrometer measurements at routine follow-up. **Results:** A total of 31 consecutive patients (15 females, 16 males; mean age 13.6 ± 1.8 years, range 9–16 years) were included. Mean follow-up was 49 ± 26 months (range 18–93 months). The mean time to return to sports was 8.8 ± 4.4 months. Eight patients (26%) experienced ACL graft rupture and underwent revision ACL reconstruction. One additional patient required partial meniscectomy. The overall revision rate was 29%. The mean subjective IKDC score was 91.8 ± 7.2, with Lysholm and KOOS scores of 96.6 ± 7.9 and 94.2 ± 5.3, respectively. No significant growth disturbances were noted. The mean side-to-side difference in KT-1000 testing was 2.2 ± 1.5 mm. Patients who underwent revision ACL reconstruction showed significantly greater length growth compared with those with intact ACL reconstruction (*p* = 0.02). Spearman correlation revealed a significant association between length growth and anterior tibial translation (*p* = 0.02, r = 0.46). **Conclusions:** Transphyseal ACL reconstruction in skeletally immature patients provides favorable clinical and radiological outcomes, with minimal risk of growth disturbance. Most patients returned to pre-injury levels of athletic activity. However, the high revision rate emphasizes the complexity of managing ACL injuries in this population.

## 1. Introduction

Anterior cruciate ligament (ACL) injuries are among the most common and significant orthopedic injuries to affect the pediatric population [1,2]. In recent years, the injury rates have risen, due to the increasing participation in competitive and high-risk sports, particularly among younger athletes [3]. These injuries can have profound long-term consequences for joint function and potential early-onset osteoarthritis and impaired mobility if not managed appropriately [4,5].

Despite advancements in ACL reconstruction (ACLR), the management of ACL deficiency in skeletally immature patients remains controversial [5,6]. A primary concern in this patient population centers on the risk of iatrogenic damage to the growth plates (physes) during surgery. This may result in complications such as leg length discrepancies or angular deformities. Given the significant remaining growth potential in these patients, it is paramount to preserve the integrity of the growth plates. Hence, clinicians must carefully balance the risks of surgical treatment with the long-term consequences of untreated ACL tears [7].

Non-operative treatment, while considered a safer and an acceptable option in some cases to avoid surgical risks, has been associated with a high incidence of secondary injuries, particularly to the meniscus and cartilage [8]. These secondary injuries are well documented in both arthroscopic evaluations and imaging studies [9,10,11]. Without surgical stabilization, ACL-deficient knees are more prone to further damage, resulting in worse functional outcomes with an increased risk of premature degenerative joint disease [12,13].

As a result of these risks, there has been a growing shift toward surgical management in pediatric patients with ACL injuries [2,5]. This trend has driven the development and refinement of various surgical techniques, including transphyseal ACL reconstruction and physeal-sparing procedures, which aim to stabilize the knee while minimizing the risk of damage to the growth plates [14]. Despite the increasing use of these methods, there remains a significant inconsistency in the literature regarding the optimal treatment algorithm for skeletally immature patients [5]. Factors that influence the choice of surgical technique include the skeletal maturity of the patient, the severity of the injury, and the surgeon’s expertise and preference [15,16].

One of the critical decisions in ACL reconstruction is the choice of graft. Various graft types are available, including patellar tendon, quadriceps tendon, and hamstring tendon, along with allografts [6,17]. Each graft has distinctive advantages and disadvantages and none is considered to be exclusively suited for pediatric patients [17]. Recent studies have shown an increasing interest in the use of quadriceps tendon autografts for ACL reconstruction in pediatric patients [18,19,20]. The quadriceps tendon offers several advantages in this population, particularly due to its larger cross-sectional diameter and lower reported graft rupture rates compared with hamstring tendon autografts. A systematic review by Rangasamy et al. reported significantly lower rupture rates in quadriceps tendon autografts compared with hamstring tendon autografts in skeletally immature patients, while also demonstrating improved functional outcomes, as reflected by higher Lysholm scores in the quadriceps tendon group [21].

Despite these promising results, hamstring tendon autografts remain the most commonly used graft for pediatric ACL reconstruction [22]. Hamstring grafts are favored due to their smaller diameter, flexibility, and lower risk of growth plate injury [23]. Unlike the patellar tendon, which can result in anterior knee pain or complications at the donor site, hamstring tendon grafts tend to show fewer associated donor site morbidities. Additionally, the relatively small size of the hamstring tendon grafts allows for easier passage through the growth plates, which in turn reduces the risk of physeal disruption [24]. Studies have also demonstrated that hamstring grafts provide adequate strength and stability while preserving knee function, while minimizing complications [25].

The primary objective of this study was to evaluate the clinical outcomes of transphyseal ACL reconstruction in skeletally immature patients, using autologous hamstring tendons as graft material. Due to its potential to provide stable knee function while reducing the risk of physeal injury, this method has gained widespread use. A secondary goal of the study was to identify risk factors that may contribute to ACL graft failure, to help improve long-term outcomes and minimize complications. Understanding these risk factors is essential to optimize surgical techniques and increase success rates of ACL reconstruction in pediatric athletes.

## 2. Methods

### 2.1. Patients and Study Design

**Figure 1:**
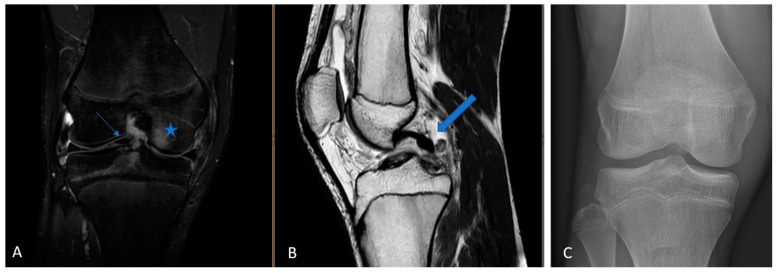
Example of a 3.0 Tesla knee MRI: (**A**) coronal PD tse fs, (**B**) sagittal T2 tse, and (**C**) conventional X-ray in AP view of 14-year-old male patient with open physis and acute ACL rupture. PD tse fs (proton density-weighted turbo spin echo sequence with fat saturation), T2 tse (T2-weighted turbo spin echo sequence), ap (anterior–posterior); large arrow is ACL rupture; star is bone marrow edema.

This retrospective study included skeletally immature patients presenting with full-thickness ACL tears, confirmed by MRI and intraoperative evaluation. Patient examples are shown in Figure 1.

The stringent inclusion criteria were as follows:

- Patients with documented open growth plates (physes) at the time of surgery.
- Full-thickness ACL tears confirmed by preoperative MRI and intraoperative assessment.
- A minimum follow-up period of 18 months to ensure adequate time to assess clinical outcomes and potential growth-related effects.
- Documented growth progression throughout the follow-up period to assess for any growth disturbances postoperatively.

Exclusion criteria included any patients with prior ipsilateral or contralateral knee injuries, significant concomitant ligament injuries, or underlying conditions that contraindicated ACL reconstruction.

The study was approved for retrospective analysis by the Charité Universitätsmedizin Berlin ethics committee (IRB No. EA2/016/21, approval date: 5 March 2021). Informed consent for data use was obtained from all patients and their guardians at the time of treatment. This consent specifically addressed the potential for anonymized data to be included in retrospective research studies, distinct from the consent provided for clinical treatment.

The sample size for this study was dictated by the rarity of ACL injuries in skeletally immature patients with open growth plates and met the specific inclusion criteria. Data collection spanned from 2005 to 2013, corresponding with the period approved by the Institutional Review Board (IRB No. EA2/016/21), granted on 5 March 2021.

### 2.2. Surgical Technique

All ACL reconstructions were performed by a single experienced orthopedic surgeon to ensure consistency in surgical technique and to minimize variability in postoperative outcomes. Surgeries were performed with patients in a supine position under general anesthesia. Standard perioperative protocols were followed, including the administration of intravenous antibiotics prior to incision. The procedure began with a diagnostic arthroscopy, conducted as part of the same surgical session immediately prior to ACL reconstruction. This diagnostic step allowed for direct visualization of injury extent and confirmation of ligament and meniscal status.

For ACL reconstruction, a four-stranded semitendinosus autograft was used as the primary graft. When necessary, an additional gracilis tendon was harvested to augment the graft. Graft harvesting was performed through a small pretibial incision, ensuring minimal disruption to the periosteum to preserve the tibial apophysis and its blood supply.

**Figure 2:**
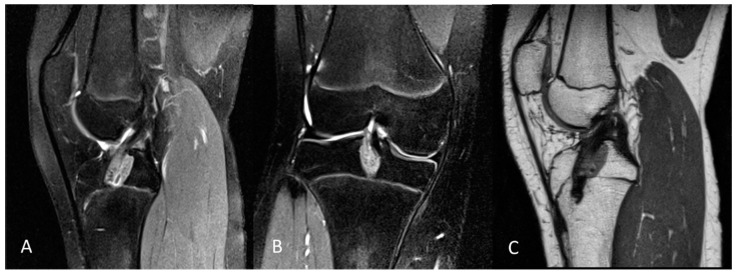
Example of a postoperative 3.0 Tesla knee MRI: (**A**) sagittal and (**B**) coronal PD tse fs, and (**C**) sagittal PD tse of a 14-year-old female patient with open physis and postoperative MRI after ACL reconstruction. PD tse fs (proton density-weighted turbo spin echo sequence with fat saturation), PD tse (proton density-weighted turbo spin echo sequence).

Tibial tunnel placement was perpendicular to the tibial physis to minimize physeal disruption. The femoral tunnel was drilled through an anteromedial portal with careful attention to avoid oblique positioning. The femoral socket was placed on the anatomical ACL footprint and drilled vertically to limit any damage to the growth plate. No osseous notchplasty was performed in each case. Graft fixation was achieved using an extracortical flip button for the femoral side, and tibial fixation was performed using a hybrid technique involving biodegradable interference screws and sutures over a bony bridge. An example of a postoperative MRI is shown in Figure 2.

In cases of meniscal instability, meniscal repair was performed using an all-inside suture technique. For patients with additional MCL instability during surgery, microperforation of the tibial attachment of the MCL was performed to stimulate a localized healing response.

### 2.3. Rehabilitation

Postoperative care involved initial immobilization of the knee in a straight splint for one week to allow early healing. After this period, the splint was replaced with a controlled-hinge orthosis that allowed for knee flexion up to 90°. The orthosis was then worn by the patient up to the fourth postoperative week. The patient was encouraged to begin isometric quadriceps contractions immediately post-surgery to maintain muscle activity. Partial weight-bearing was allowed from the first week onward, along with gradual passive mobilization of the knee joint. After the fourth week, the patient progressed to an unrestricted passive range of motion exercises and full weight-bearing as tolerated, with the initiation of an active range of motion exercises.

A return to competitive sports activities, particularly those involving cutting, pivoting, or jumping, was permitted from the ninth postoperative month, contingent upon ligamentous stability, which was determined by clinical assessment.

### 2.4. Clinical Evaluation

At follow-up, a comprehensive physical examination of both knees was performed by an independent musculoskeletal physician who was not involved in the surgical procedures. Collected data included patient demographics, anthropometric measurements (height, weight), concomitant injuries, and treatment specifics. The body mass index (BMI) was calculated both at the time of surgery and at the final follow-up (BMI = weight in kg/height in m²).

Clinical outcomes were evaluated using several standardized instruments, including the subjective and objective International Knee Documentation Committee (IKDC) knee ligament evaluation form, the Lysholm knee scoring scale, and the knee injury and osteoarthritis outcome score (KOOS). Assessment of growth disturbances and leg length discrepancies was performed through clinical measurements, using tape to measure the distance from the anterior superior iliac spine to the lateral malleolus, supplemented by block placement techniques for precise evaluation.

Knee stability was assessed using the Lachman test and the pivot shift test, with anterior tibial translation quantified using a KT-1000 arthrometer (MEDmetric Corp., San Diego, CA, USA). The maximal anterior displacement of the injured knee was compared with that of the contralateral uninjured knee.

### 2.5. Statistical Analysis

Data analysis was performed using Prism Version 6 (GraphPad Software Inc., San Diego, CA, USA). Continuous variables were expressed as mean ± standard deviation (SD) or median [interquartile range], depending on the distribution of the data. The D’Agostino and Pearson omnibus normality test was used to assess the Gaussian distribution of variables. Comparisons of parametric data were conducted using the Student’s *t*-test, while non-parametric data were analyzed using the Mann–Whitney U test. Correlations between variables were assessed using Spearman’s correlation coefficient.

A *p*-value of <0.05 was considered statistically significant for all tests performed.

## 3. Results

### 3.1. Patients

A total of 31 patients (15 females, 16 males; mean age 13.6 ± 1.8 years) were included. The mean time from injury to surgery was 5.8 months (range 0–36 months), with 18 patients (58%) undergoing surgery within 12 weeks (mean 8.4 ± 2.2 weeks). Mean preoperative BMI was 22 ± 3.7 kg/m² (Table 1).

### 3.2. Clinical Evaluation Outcomes

**Figure 3:**
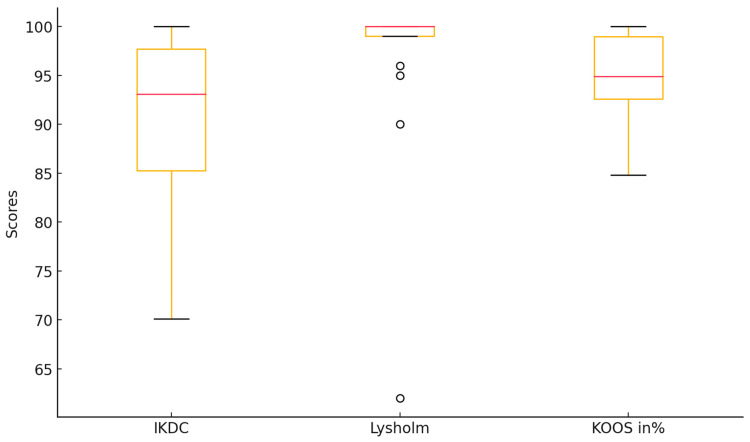
Visualization of the distribution of IKDC, Lysholm, and KOOS scores, showing predominantly good to very good results. While the IKDC score displays some variability, the Lysholm and KOOS scores are more consistently clustered near the upper range.

A high rate of favorable functional outcomes was observed, with a mean subjective IKDC score of 91.8 ± 7.2, Lysholm score of 96.6 ± 7.9, and KOOS score of 94.2 ± 5.3. Additionally, knee stability assessments indicated a mean side-to-side difference of 2.2 ± 1.5 mm in KT-1000 testing, with no significant difference between patients undergoing primary versus revision ACL reconstruction (Figure 3).

### 3.3. Revision Rate and Graft Failure

During the follow-up period, eight patients (26%) experienced ACL graft failure and underwent revision ACL reconstruction. One additional patient required partial meniscectomy. The overall revision rate was 29%. There were no significant differences in the IKDC, Lysholm, or KOOS scores between patients undergoing primary versus revision ACL reconstruction, suggesting that functional outcomes following revision surgery were comparable to those after primary surgery.

### 3.4. Knee Stability and Growth-Related Changes

The mean side-to-side difference in KT-1000 arthrometer testing was 2.2 ± 1.5 mm, indicating satisfactory knee stability in the majority of patients. There was no statistically significant difference in side-to-side laxity between patients with primary vs. revision ACLR, although a slight increase in laxity was noted in the revision ACLR group (1.9 ± 1.4 mm vs. 2.9 ± 1.5 mm; *p* = 0.12).

### 3.5. Anthropometric Assessment

Patients showed expected growth during the follow-up period. The mean BMI increased from 22.3 ± 3.7 kg/m² preoperatively to 23.9 ± 5.1 kg/m² at final follow-up (*p* = 0.21), reflecting normal growth patterns. No significant correlation was found between BMI and any of the primary clinical outcomes.

The mean increase in patient height from surgery to final follow-up was 7 cm (range 2–32 cm). A significant difference in growth was observed between patients who required revision ACL reconstruction and those who did not. Patients who underwent revision ACLR had an average height increase of 12 ± 8.7 cm, compared with 5 ± 4.3 cm in patients without graft failure (*p* = 0.02). There was also a significant association between increase in height and anterior tibial translation, as measured by KT-1000 testing (*p* = 0.02, r = 0.46), suggesting that continued growth may influence postoperative knee stability.

## 4. Discussion

This study aimed to evaluate the clinical outcomes of transphyseal ACL reconstruction in skeletally immature patients. Our findings indicate that transphyseal ACL reconstruction in this population yields favorable clinical outcomes without substantial growth disturbances. The majority of patients returned to preoperative levels of athletic activity, and no significant leg length discrepancies or angular deformities were observed. However, a notable finding was the high revision rate of 26%, with a strong correlation between residual length growth post-surgery and ACL graft failure.

The patient age range in our study reflects the typical demographic for pediatric and adolescent populations undergoing ACL reconstruction (ACLR), a population that often presents unique challenges related to growth potential and activity levels [12,26].

Notably, 58% of the patients underwent surgery within the first twelve weeks post-injury, which suggests a trend toward early surgical intervention in the majority of the cohort. This early intervention is important, as previous research has shown that delays in surgical treatment can be associated with an increased risk of secondary injuries, such as meniscal or chondral damage [27]. However, the wide range of time to surgery indicates a variability in clinical decision making, which may be influenced by factors such as patient preference, severity of injury, or access to care.

Our study did not show a significant correlation between BMI and primary clinical outcomes; however, it is important to note that our sample size of 31 patients was relatively small, especially compared with larger studies in the literature that have reported on the relationship of BMI and ACLR outcomes in larger cohorts [28]. The limited sample size in this study restricts the generalizability of our findings regarding BMI and may underpower the detection of potential associations between BMI and surgical outcomes. Future studies with larger sample sizes are needed to more conclusively examine the influence of BMI on outcomes in younger skeletally immature patients.

A majority of patients (90%) achieved good or very good results with the objective IKDC assessment. This finding aligns with previous studies that showed a majority of pediatric patients with ACLR recover to near-normal levels of function [13]. The Lysholm score, which focuses on knee function and stability, yielded a mean of 96.6 ± 7.9 points, further supporting the positive functional outcomes of the cohort. Additionally, the knee injury and osteoarthritis outcome score (KOOS) was 94.2 ± 5.3%, again showing excellent knee function and a high level of satisfaction among patients. There was no significant difference between primary and revision ACLR for either the Lysholm score or KOOS, suggesting that revision surgery can achieve outcomes comparable to primary reconstruction in this cohort.

It is also notable that no clinical evidence of aberrant growth, including leg length discrepancies (≥5 mm) or angular deformities, was observed in this cohort. This is a particularly important finding given the age of the patients and their ongoing growth at the time of surgery.

The mean maximum side-to-side difference in instrumented KT-1000 testing was 2.2 ± 1.5 mm, indicating satisfactory ligament stability in most patients. There was no significant difference in side-to-side laxity between patients who underwent primary ACLR and those who required revision ACLR (1.9 ± 1.4 vs. 2.9 ± 1.5 mm; *p* = 0.12). This result further underscores the success of revision ACLR in restoring knee stability to a level similar to that of primary reconstruction, although the slight increase in laxity in the revision group may suggest some residual ligamentous laxity [29].

In our study, we observed an increase in BMI from preoperative to final follow-up, although this change was not statistically significant (*p* = 0.21). Due to the small sample size of 31 patients, these findings should be interpreted with caution, as the study may lack sufficient power to detect a meaningful association between BMI and surgical outcomes. This absence of a significant association may indicate that other factors, such as age-related growth rates, play a more influential role in graft stability during development [28,30,31].

Larger longitudinal studies are needed to assess BMI’s impact on ACL reconstruction outcomes in pediatric and adolescent patients. Research on BMI’s risks and benefits during developmental years could clarify its role in recovery and stability after ACL reconstruction in youth [32].

Patients who underwent revision ACLR exhibited significantly greater growth during follow-up compared with those with intact ACLR (12 ± 8.7 cm vs. 5 ± 4.3 cm; *p* = 0.02). This finding underscores the importance of monitoring growth in young ACLR patients, as rapid or uneven growth may increase stress on the graft, potentially leading to graft rupture. A significant correlation was observed between height increase and the side-to-side difference in anterior tibial translation (*p* = 0.02, r = 0.46), suggesting that growth may impact ligament stability post-operatively. This relationship between growth and knee laxity highlights the need for further research on growth patterns and ACL stability to improve surgical and rehabilitation protocols for pediatric patients [32].

There was no significant difference in age at the time of surgery between patients who required revision ACLR and those who did not (13 ± 2 years vs. 14 ± 1 years; *p* = 0.23). This indicates that age alone is not a determining factor for the risk of graft rupture or revision surgery in this population.

The high revision rate in our cohort aligns with previous studies, which reported significantly increased revision rates in skeletally immature patients compared with adults, even with recent advancements in surgical techniques [33,34]. Our study reinforces this finding and adds to the existing body of the literature that the primary risk factor for graft failure is not the patient’s age at the time of surgery but rather the amount of growth that occurs after the procedure. Specifically, patients with significant residual growth following surgery are more likely to experience graft failure compared with those nearing skeletal maturity.

Our findings align with other studies that report favorable functional outcomes following transphyseal ACL reconstruction in skeletally immature patients, with high IKDC, Lysholm, and KOOS scores and minimal growth disturbances [32]. The revision rate in our cohort (26%) is also in line with previous research that suggest an increased risk of graft failure in pediatric populations, likely owing to ongoing growth and the physical demands of returning to high-impact activities [35,36].

Current rehabilitation protocols are largely adapted from adult regimens, yet research suggests that adolescents may not regain functional movement patterns as effectively as adults [37]. Boyle et al. reported that functional recovery in adolescents following ACL reconstruction is often incomplete when compared with adult patients [30]. This observation highlights the need for rehabilitation strategies tailored to skeletal maturity that take into account the ongoing skeletal development of pediatric patients. Adapting rehabilitation programs to accommodate the unique challenges of skeletal immaturity may help reduce the incidence of graft failure and improve long-term outcomes.

The risk of physeal injury remains a major concern in pediatric ACL reconstruction, given the potential for growth disturbances resulting from tunnel placement or graft fixation across the growth plate. However, our study confirms that transphyseal drilling, when performed with careful and particular attention to physeal anatomy, is a safe and effective approach, which is in line with the existing literature [32]. No substantial growth disturbances, such as leg length discrepancies or angular deformities, were observed in our cohort. These findings underscore the safety of this technique when performed by experienced surgeons [31,38]. Makela et al. described that growth disturbances occur when more than 7% of the cross-sectional area of the physis is damaged [39]. Our results, consistent with prior studies, show that with meticulous surgical technique, transphyseal ACL reconstruction does not lead to significant physeal injury.

This study has several noteworthy limitations. A primary limitation is its retrospective nature, which inherently limits the ability to control for all potential confounding factors. Additionally, we made a deliberate decision to exclude preoperative MRI scans from our analysis. This exclusion was largely driven by the poor quality of many available scans and the inaccessibility of digital data sets in most cases. Consequently, we were unable to consider or analyze potential degenerative changes that might have occurred during the progression of anterior cruciate ligament reconstruction. While this could be seen as a limitation, it does not impact the interpretation of our findings, which focus primarily on postoperative clinical outcomes and functional recovery.

Another limitation is attributable to the fact that ACL injuries are uncommon in patients with open physes. Hence, we included a broad spectrum of concomitant injuries within the patient cohort with ACL injuries. On the one hand, this approach increased the scope of our analysis, but, on the other hand, it also introduced the possibility of additional confounding factors attributable to concomitant injuries. To mitigate for potential confounding factors and bias, we used stringent inclusion criteria.

Despite these challenges, our findings provide valuable clinical insights into a rare injury in the pediatric population.

## 5. Conclusions

ACL reconstruction in patients with open physes yields favorable clinical outcomes, albeit with a higher-than-expected revision rate. Importantly, transphyseal tunnel placement did not lead to growth abnormalities in our cohort. Finally, the risk of ACL graft failure was not primarily influenced by patient age but rather by remaining growth potential. This emphasizes the need for careful consideration of growth potential when managing ACL injuries in skeletally immature patients.

## Figures and Tables

**Table 1:** Aggregated demographic patient data.

Variable	Measure	31 Patients
Age (y)	Mean (SD)	13.6 (1.8)
	Range	9–16
Body Mass Index	Mean (SD)	22.0 (3.7)
Gender (males)	n (%)	16 (52%)
Meniscus Repair	n (%)	1 (3%)
ACLR-Failure and Revision	n (%)	8 (29%)
Time from Injury to Surgery (months)	Mean (SD)	5.8 (7.3)

## Data Availability

The original contributions presented in this study are included in this article, further inquiries can be directed to the corresponding author/s.
